# Effect of Monocerin, a Fungal Secondary Metabolite, on Endothelial Cells

**DOI:** 10.3390/toxins15050344

**Published:** 2023-05-18

**Authors:** Tainah Colombo Gomes, Rafael Conrado, Rodrigo Cardoso de Oliveira, Priscila Jane Romano Gonçalves Selari, Itamar Soares de Melo, Welington Luiz Araújo, Durvanei Augusto Maria, Ana Olívia De Souza

**Affiliations:** 1 Development and Innovation Laboratory, Instituto Butantan, Avenida Vital Brasil, 1500, Sao Paulo 05503-900, SP, Brazil; tainahcolombogomes@gmail.com (T.C.G.); rafael.conrado@butantan.gov.br (R.C.); durvanei.maria@butantan.gov.br (D.A.M.); 2 Department of Biochemical and Pharmaceutical Technology, FCF, University of Sao Paulo, Sao Paulo 05508-000, SP, Brazil; 3 Federal Institute of Education, Science and Technology Goiano, Ceres 76300-000, GO, Brazil; priscila.goncalves@ifgoiano.edu.br; 4 Environmental Microbiology Laboratory, EMBRAPA Meio Ambiente, Jaguariuna 13918-110, SP, Brazil; 5 Laboratory of Molecular Biology and Microbial Ecology (LABMEM), Microbiology Department, ICB II, University of Sao Paulo, Sao Paulo 05508-000, SP, Brazil; wlaraujo@usp.br

**Keywords:** secondary metabolite, monocerin, cell viability, cell integrity, proliferation, senescence

## Abstract

This study reports the isolation and identification of the endophytic fungus *Exserohilum rostratum* through molecular and morphological analysis using optical and transmission electron microscopy (TEM), as well as the procurement of its secondary metabolite monocerin, an isocoumarin derivative. Considering the previously observed biological activities of monocerin, this study was performed on human umbilical vein endothelial cells (HUVECs) that are widely used as an in vitro model for several different purposes. Important parameters, such as cell viability, senescence-associated β-galactosidase, cellular proliferation by using 5(6)-carboxyfluorescein diacetate *N*-succinimidyl ester (CFSE), apoptosis analysis with annexin, cellular morphology through scanning electron microscopy (SEM), and laser confocal analysis were evaluated after exposing the cells to monocerin. After 24 h of exposure to monocerin at 1.25 mM, there was more than 80% of cell viability and a low percentage of cells in the early and late apoptosis and necrosis. Monocerin increased cell proliferation and did not induce cell senescence. Morphological analysis showed cellular integrity. The study demonstrates aspects of the mechanism of action of monocerin on endothelial cell proliferation, suggesting the possibility of its pharmaceutical application, such as in regenerative medicine.

## 1. Introduction

Natural products are relevant for drug discovery and can be found in plants, microorganisms, animals, or marine organisms [1,2]. Mainly, microorganisms have been widely used for different biotechnological applications. Since the discovery of penicillin, fungi have been widely explored as a source of new compounds by the academy and pharmaceutical industries [1,2].

Brazil has a vast territory with immense biodiversity, and its biome *Caatinga* is uniquely interesting for research in drug discovery due to its environmental conditions. The temperature of this biome, around 25–29 °C during the year, and the relative air humidity, about 50%, influence a specific vegetal formation. In a drug discovery study, the endophytic fungus *Exserohilum rostratum* was isolated from the leaves of *Croton blanchetianus*, an endemic plant from this biome. The secondary metabolite monocerin was obtained through the fungus culture and physically and chemically characterized, as previously reported [3].

Monocerin is an isocoumarin derivative, classified as a polyketide metabolite, which has been isolated from several species of fungi. The literature data show that monocerin was already obtained from *Helminthosporium monoceras* (*Dreschlera monoceras*) [4], *Exserohilum turcicum* (*Dreschslera turcica*) [5], *Fusarium larvarum*, *Dreschlera ravenelii, Microdochium bolleyi* [6], from *E. rostratum* [3,7], and, lately, from *Penicillium chrysogenum* SCSIO 41001 [8]. Among these species, *E. rostratum* has been described as a phytopathogen; however, an outbreak of fungal meningitis associated with contaminated methylprednisolone injections was reported by the Center of Diseases Control (CDC) in the United States in 2012 [9], culminating in the death of 8% of patients after 12 months of the diagnostic [10]. Cases of sinusitis [11] and keratitis [12] were also reported. Lately, this species has been successfully used as a model of rabbit meningoencephalitis [13].

Despite the reported infections, mainly in immunocompromised patients, several studies have shown the potential of this species as a source of bioactive secondary metabolites [14,15,16,17]. Among them, previous studies showed a diversified spectrum of biological activities of monocerin [3,18,19]. In this regard, its insecticidal effect was already described [20], as well as its phytotoxicity [21], antiplasmodial activity against the multidrug-resistant K1 strain of *Plasmodium falciparum* (IC50 value of 0.68 μM) [3], antimicrobial [7], and its cytotoxicity against L5178Y cells, a murine lymphoma cell line, with an IC50 of 8.4 μM [8].

Considering the previously observed wide spectrum of biological activities of monocerin, this manuscript describes its effect at the cell level on endothelial cells (HUVECs), in order to explore and open new possibilities of application, such as by the pharmaceutical industry.

Endothelial cells are essential for homeostatic events, such as inflammations, and are engaged in repairing damaged tissues through biochemical processes triggered mainly by cellular migration, proliferation, differentiation, and secretory function that can also repair damaged blood vessels [22,23]. Endothelial cells are used as in vitro models for different purposes, such as in regenerative medicine [24,25,26]. To this intent, the cell viability, proliferation, senescence, apoptosis (annexin V/propidium iodide (PI)), and morphology through SEM and laser confocal analysis through acridine orange and rhodamine 123 staining were evaluated on HUVECs.

## 2. Results

### 2.1. Identification and Morphological Characterization of the Fungal Isolate

**Figure 1:**
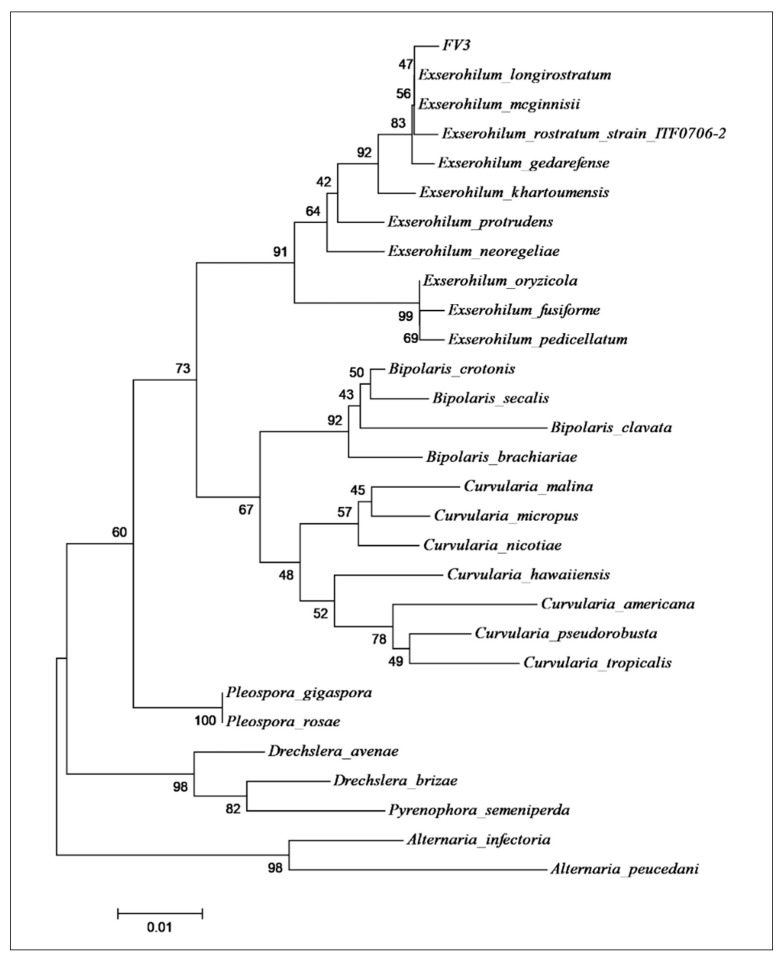
Phylogenetic tree built by the neighbor-joining method using the Jukes and Cantor models for the ITS1-5,8S-ITS2 sequence of the endophytic strain FV3 used to produce the compound monocerin. The GenBank accession of the sequences used in this analysis is presented in Supplementary Material (Table S1).

The taxonomic identification of the FV3 fungal isolate was based on the phylogenetic tree analysis using the ITS1-5,8S-ITS2 sequences compared to the nucleotide sequence from the NCBI database number MK005195 (GenBank) (Table S1). The query sequence of the fungus presented 97% of identity with *E. rostratum,* and the evolutionary distances, computed using the Jukes-Cantor method, show that the strain FV3 is close to *E. rostratum, E. longirostratun,* and *E. mcginnisii* (Figure 1).

According to Hernández-Restrepo et al. [27], these *Exserohilum* species are molecularly very closely related and should be treated as synonyms; however, they can be morphologically highly variable. According to the data, the FV3 isolate was identified as *E. rostratum* (Figure 1), and it was deposited at the Adolfo Lutz Institute Collection (IAL 7247) (http://www.wfcc.info/ccinfo/collection/by_id/282 accessed on 3 February 2023; http://www.ial.sp.gov.br/ accessed on 3 February 2023), São Paulo, Brazil.

**Figure 2:**
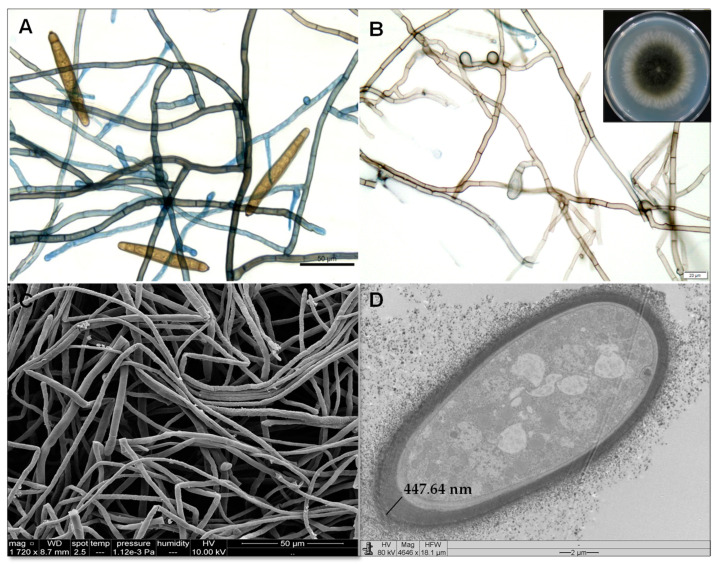
Morphological features of the fungus *E. rostratum* obtained through optical microscopy ((**A**,**B**), bars of 50 and 20 µm), including an inset of the fungus colony picture (**B**), scanning electron microscopy ((**C**), 1720×, bar = 50 µm), and transmission electron microscopy ((**D**), 4646×, bar = 2 µm). Indication of the thickness wall, containing melanin, measured using TEM as 447.64 nm (Leo 906 E Zeiss microscopy serial # 9682, Kv maximum of 120 Kv).

The morphological analysis of the FV3 isolate performed by SEM and TEM indicated that its characteristics are in accordance with the *E. rostratum* species description [27]. Through the macro and micromorphology analysis shown in Figure 2, colonies display hairy to cottony and olivaceous black mycelium with a fimbriate margin. The hyphae are septate, pale olivaceous to pale olivaceous brown, and the conidia are fusiform, olivaceous brown, and distoseptate (*n* = 7), with a protruding hilum.

*E. rostratum* is a thermophilic pigmented mold that thrives in warm climates and is usually found in soil and plants, especially grasses [28]. This species is classified as a dematiaceus fungus due to the presence of melanin pigments [29], whose aromatic rings contribute to reflecting and capturing of the light and protection of microorganisms against the damage caused by radiation. This pigment is synthesized and deposited near the cell wall or secreted to the extracellular environment [29]. However, its accumulation in the cell wall confers surviving advantages to dematiaceus fungi in extreme environments [30], such as thermoregulation, photoprotection, antioxidant action, mechanical resistance, and protection against dehydration.

The TEM analysis allowed us to observe and determine the thickness of the melanin layer in the *E. rostratum* cell wall (Figure 2). Hence, is it plausible that the high amount of melanin in its cell wall is associated with its extremophilic characteristics, supporting high temperatures in the environment.

### 2.2. Fungus Culture and Obtaining of Monocerin

Monocerin was produced by the fungus, and, after extraction from the culture supernatant and purification by HPLC, its degree of purity was confirmed by GC/MS. (Figure S1). A culture of 500 mL yielded 16 mg of the pure compound, which was physically and chemically characterized, as previously demonstrated [6,7,15], according to the data below.

Monocerin (IUPAC): (2S,3aR,9bR)-6-hydroxy-7,8-dimethoxy-2-propyl-2,3,3a,9b-tetrahydro-5H-furo[3,2-c]isochromen-5-one.

^1^H NMR (499.8 MHz, CDCl_3_): δ 11.17 (s, OH), 6.85 (s, 1H), 5.20 (dd, 6.0 and 3.2 Hz, 1H), 4,6 (d, 3.2 Hz, 1H), 4.00 (m, 1H), 3.89 (s, OCH_3_), 3.71 (s, OCH_3_), 2.60 (ddd, 14.5, 8.4 and 6.0 Hz, 2H), 1.90 (dd, 8.4 and 6.0 Hz, 2H), 1.47 (m, 2H), 1.26 (m, 2H), 0.84 (t, 7.3 Hz, 3H); ^13^C NMR (125.7 MHz, CDCl_3_): δ 168.1, 158.8, 155.3, 136.7, 132.4, 105.7, 102.0, 82.0, 77.9, 73.9, 60.4, 56.8, 38.8, 38.3, 19.1, 14.4; electron ionization *mass spectra* (EIMS) *m/z*, 308.1 [M]^+^, 265.1, 247.0, 221.0, 209.0, 167.0, 148.0, 81.0.

### 2.3. Cell Viability

**Figure 3:**
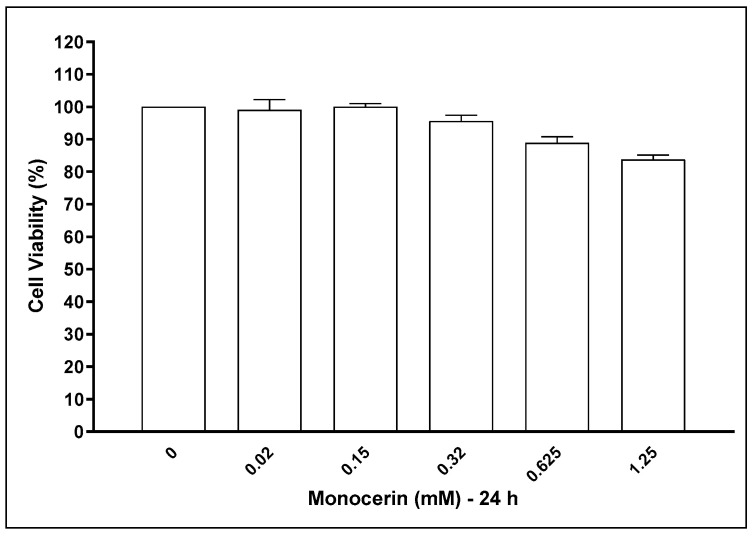
Representation of endothelial cells’ (HUVECs) viability after treatment with monocerin from 0.02 to 1.25 mM for 24 h in six replicates in three independent assays (mean ± SE). The control group refers to untreated cells that receive only serum-free RPMI-1640 medium and were set as 100% of cell viability.

Figure 3 represents the viability of HUVECs treated with monocerin for 24 h. At 0.625 and 1.25 mM there was 88.89 and 83.73% of cell viability, indicating, in comparison with the control group, a decrease of 11.11 and 16.77%. However, these decreases were not statistically significant.

### 2.4. Proliferation Index by CFSE Assay

The carboxyfluorescein succinimidyl ester CFSE assay quantifies the cell proliferation index according to the fluorescence intensity that is related to the cell number.

**Figure 4:**
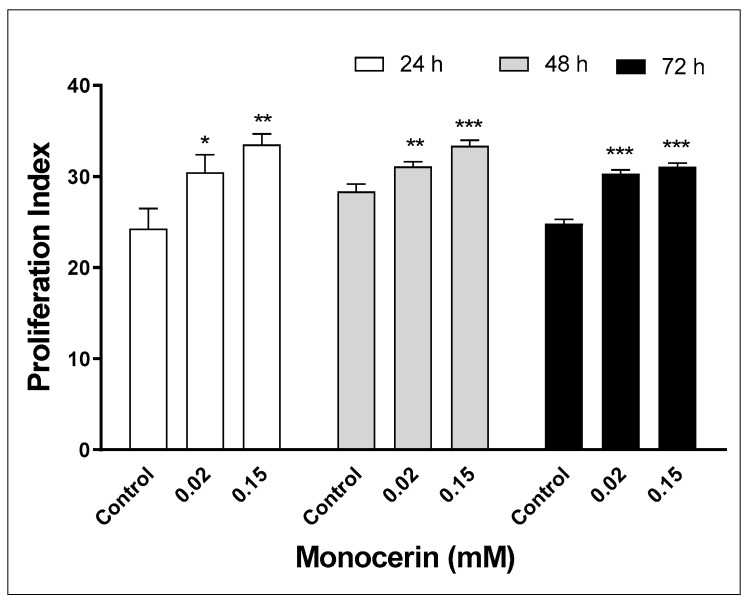
Proliferation index of endothelial cells (HUVECs) exposed to monocerin at 0.02 and 0.15 mM in triplicate for 24, 48, and 72 h. Data represent mean ± SE from two independent experiments compared to untreated cells (control). * *p* < 0.05. ** *p* < 0.01 and *** *p* < 0.001.

As can be observed in Figure 4, there was a statistically significant increase in the proliferation indexes of HUVECs exposed to monocerin from 24 to 72 h. At 24 h, the proliferation indexes were 24.3 ± 3.1, 30.4 ± 2.7, and 33.5 ± 1.6 for control and monocerin at 0.02 and 0.15 mM, respectively. In comparison with the control, at 0.02 and 0.15 mM, monocerin induced an increase of 1.25 and 1.38 times for 24 h. For the control and monocerin at 0.02 and 0.15 mM, the proliferation indexes were 28.3 ± 1.2, 31.1 ± 0.7, and 33.4 ± 0.9 for 48 h, and 24.8 ± 0.7, 30.3 ± 0.6, and 31.1 ± 0.6 for 72 h, respectively. At 48 and 72 h, there was an increase, mainly at 0.15 mM.

### 2.5. Annexin V/PI Labeling of Endothelial Cells Treated with Monocerin

**Figure 5:**
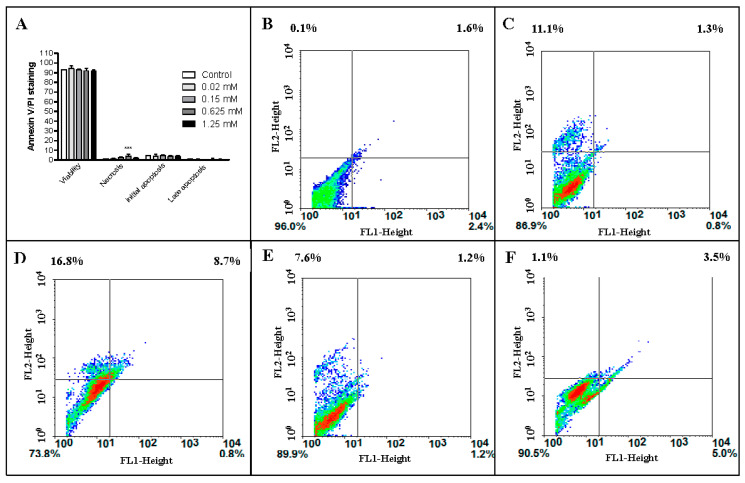
(**A**) Percentage of viable cells, in necrosis, and in initial and late apoptosis (mean ± SE). Histogram of double annexin/PI staining of untreated (control) endothelial cells (**B**) and following the treatment with monocerin at (**C**) 0.02, (**D**) 0.15, (**E**) 0.625, and (**F**) 1.25 mM. Quadrants: left lower = viable cells; right lower = apoptotic cells (annexin V+); upper right = late apoptotic cells (annexin V+ and PI+); upper left = necrotic cells (PI+). Cell distribution in viable, apoptotic (annexin V+), late apoptotic (annexin V+ and PI+), and necrotic cells (PI+). (*** *p* < 0.001).

The annexin V corresponding signal is a very sensitive method for detecting cell apoptosis, whereas PI is used to detect necrotic or late apoptotic cells, characterized by the loss of plasma and nuclear membranes’ integrity. The data presented in Figure 5 and Table 1 show a high percentage of viable cells, even when treated with monocerin at 1.25 mM.

### 2.6. Endothelial Cell Senescence—β-Galactosidase Activity

**Figure 6:**
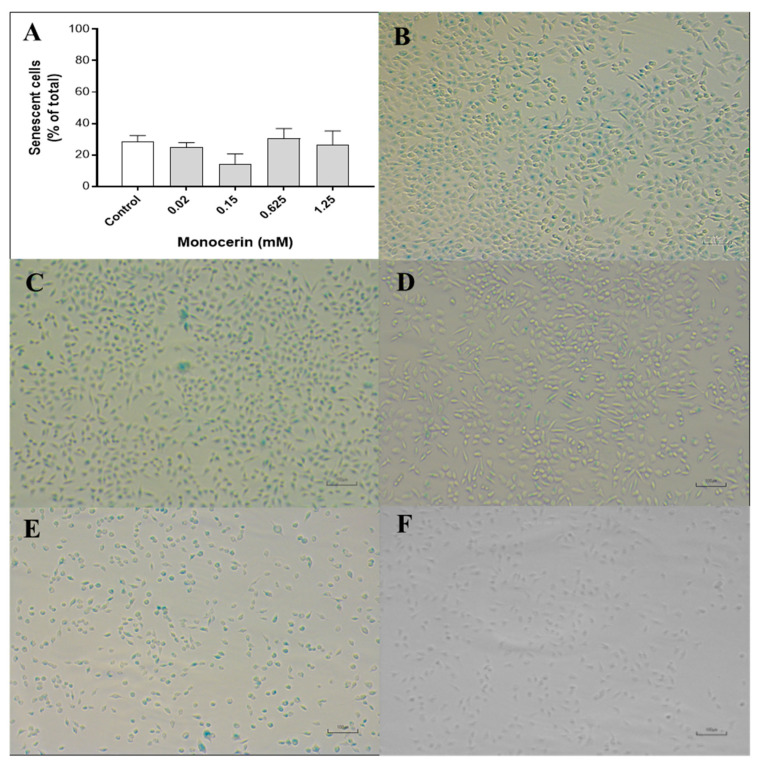
(**A**) Percentage of senescence of endothelial cells treated with monocerin (Mean ± SE), as well as photomicrograph representatives of senescence processes for (**B**) untreated (control) cells and those treated with monocerin at (**C**) 0.02, (**D**) 0.15, (**E**) 0.625, and (**F**) 1.25 mM for 24 h. Cells in blue are senescent, and the brilliant colorless ones are non-senescent cells detected on the β-galactosidase assay. Bars = 100 µm.

Figure 6A–F illustrates the cytochemical staining of the endothelial cells after the treatment with monocerin for 24 h. At 24 h, the percentage of positively SA-β-gal-stained cells for the control was 28.6 ± 3.6%, and, for monocerin at 0.02, 0.15, 0.625, and 1.25 mM, the percentages were 24.9 ± 2.8, 14.3 ± 6.2, 30.6 ± 6.2, and 26.6 ± 8.5%, respectively. There was no statistical difference in the percentage of senescence among control and treatments with monocerin for all evaluated concentrations.

### 2.7. Morphological Analysis of the Endothelial Cells

**Figure 7:**
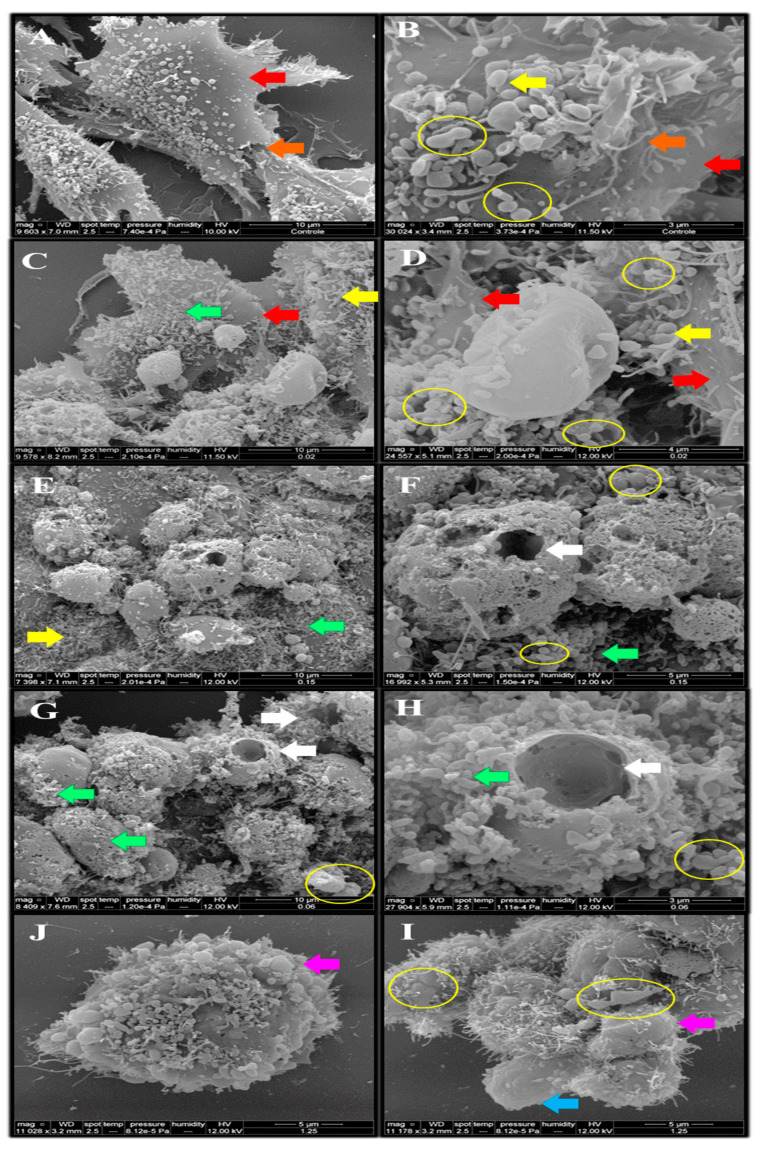
Photomicrograph obtained through scanning electron microscopy of untreated (control) endothelial cells (**A**,**B**) (10 μm/9603× and 3 μm/30,024×), and under exposure to monocerin for 24 h at (**C**,**D**) 0.02 (10 μm/9578× and 4 μm/24,557×), (**E**,**F**) 0.15 (10 μm/7398× and 5 μm/16,992×), (**G**,**H**) 0.625 (10 μm/8409× and 3 μm/27,904×), and (**I**,**J**) 1.25 mM (5 μm/11,178× and 5 μm/11,028×). Control cells were adherent, willing to constitute a monolayer with the presence of extracellular matrix formation (red arrows), cytoplasmic prolongations, and adhesion to the surface (yellow arrows). Cell junction elements and microvillus are present (orange arrow). Granules of different sizes and forms are adhered and distributed around the cellular body surface for the control and the monocerin group (green arrow). HUVECs treated with monocerin showed retractions of their cytoplasms and inter-cell communication due to the induction of cell division (yellow circle). For monocerin at 0.625 mM, there is an apoptotic body in degeneration, showing holes with corrosive aspect (white arrow). For the higher concentration of 1.25 mM of monocerin, the cell adhesion was lost (blue arrow), and a loss of the microvillus can be observed. There is formation of cellular aggregates, apoptotic bodies (pink arrows), and bubbles around the membrane surface.

As can be observed in Figure 7, SEM shows that untreated cells (control) were adherent with the presence of extracellular matrix, cytoplasmic prolongations, cell junction elements, and microvillus. The cell morphology after treatment with monocerin was preserved mainly at 0.02, 0.15, and 0.625 mM. This result is in accordance with those found by annexin V/PI analysis. There are several cells in division, and the presence of granules of different sizes and forms adhered and distributed around the cell body surface is observed.

**Figure 8:**
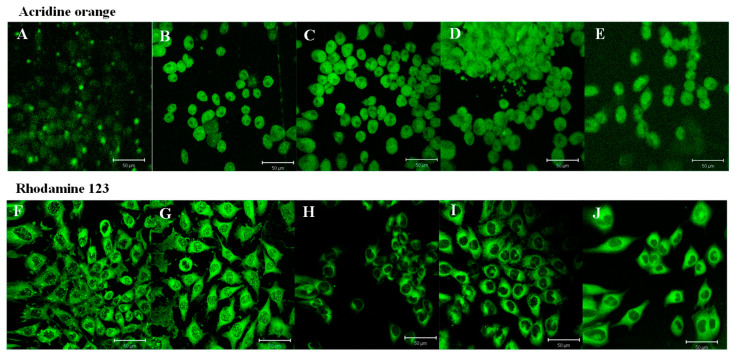
Photomicrographs obtained through laser confocal microscopy of untreated (control) endothelial cells (**A**) and treated with monocerin at (**B**) 0.02, (**C**) 0.15, (**D**) 0.625, and (**E**) 1.25 mM for 24 h and labeled with acridine orange, an indicator for acidic vacuoles in cells and lysosomes. For treatments with 0.02, 0.15, and 0.625 mM of monocerin, it is possible to observe cell division. For cells treated with monocerin, mainly at 0.625 mM, there is a nuclear modification with heterogeneous chromatin distribution and aggregation of DNA in the nucleus with lower fluorescence intensity. At 1.25 mM, there is disorganization of the nuclear material, with significant loss of cell density and prominences on the surface of the cell membrane and dead cells. Illustration for rhodamine 123 staining for (**F**) untreated cells, or treated with monocerin at (**G**) 0.02, (**H**) 0.15, (**I**) 0.625, and (**J**) 1.25 mM. Rhodamine staining shows homogeneous distribution of mitochondria in the cytoplasm. At 0.625 and 1.25 mM, there is nuclear pleomorphism and retraction of the cytoplasmatic expansions. Bars = 50 µm.

Although the percentage of necrotic cells due to treatment with 0.625 mM was very low, the presence of an apoptotic body was observed with SE (Figure 8). When treated with monocerin at the higher concentration of 1.25 mM, the cell adhesion was lost, and microvillus loss can be observed. There is a presence of cellular aggregate formation, apoptotic bodies, and bubbles around the membrane surface.

The morphology of HUVECs stained with AO or rhodamine 123 was analyzed to gain more insight into the cell viability after treatment with monocerin (Figure 8). The results showed that, for the control cells, the nuclei did not change, and the DNA remained intact, as well as the cell membrane, indicating the cell viability. It is possible to observe the presence of mitochondria around the nucleus and cytoplasm compartment.

The acridine orange stain is used to visualize and quantify nucleic acids in cells. This dye binds to DNA by intercalation, and, when it binds to double-stranded DNA, a green fluorescence is emitted. In the endothelial cells treated with monocerin at 0.625 and 1.25 mM, there are aggregates of DNA within the nucleus, as well as a decreased intensity of green fluorescence and cell debris (Figure 8). The treatment with 1.25 mM showed a homogeneous distribution of heterochromatin and a decrease in cell density, as observed when analyzed through SEM. At the lower concentrations of 0.02 and 0.15 mM, DNA aggregates are not observed.

For the cells treated with monocerin at 0.02, 0.15, 0.625, and 1.25 mM and stained with rhodamine 123, a homogeneous mitochondrial distribution was observed in the cytoplasm (Figure 8). At 0.625 and 1.25 mM, monocerin induced nuclear pleomorphism and retraction of the cytoplasmatic expansions, evidenced by bubbles formed around the cellular membrane and decrease fluorescence in the cytoplasmic compartment.

## 3. Discussion

The integrity and normal function of endothelial cells are essential for homeostatic events [22,31]. Therefore, the investigation on the mechanism of cellular proliferation induced by monocerin on this cell line is important to elucidate its effect. In this study, in addition to the classical cytotoxicity assay by 3-(4, 5-dimethyl thiazolyl-2)-2, 5-diphenyltetrazolium bromide (MTT) analysis, the annexin V/PI staining allowed the differentiation of cells in necrosis, as well as in initial and late apoptosis, and showed that the percentage of viable cells was higher than 90%, even after the treatment with monocerin at the very high concentration of 1.25 mM. The percentage of cells in necrosis or apoptosis was not substantial. Therefore, our results show that monocerin protects endothelial cells from death.

CFSE analysis allows direct detection of cell proliferation by flow cytometry. Hence, in a whole-cell population undergoing proliferation, fluorescence intensity declines by half in the following generation [31].

Cellular senescence is part of tightly orchestrated biological processes in several cell functions such as embryonic development, tumor suppression, wound healing, and tissue repair [32,33,34,35,36]. Senescence represents a series of progressive and phenotypically diverse cell states acquired after the initial growth arrest. This challenge arises from the fact that cell proliferation is essential for regeneration and repair, and, hence, is essential for the health of organisms with renewable tissues.

Senescent cells remain metabolically active and often acquire an altered transcriptional profile. An irreversible loss of cell proliferation potential occurs in the senescence process, along with a super expression of acidic lysosomal β-galactosidase. In general, the growth stops, and usually, the cell can increase up to double its size. There is a cytoplasmic prolongation, similar to mesenchymal cells. When adherent, the cell morphology is modified. Usually, a multinucleated and/or irregular cell nuclei and chromatin reorganization can be observed, showing a high number of granules [37,38]. Interestingly, these events were not observed in endothelial cells treated with monocerin and analyzed using SEM and confocal analysis by AO and rhodamine staining. The induction of senescence in endothelial cells due to monocerin treatment for all assayed concentrations was not different from that of control (untreated cells). These characteristics support the important role of monocerin in the equilibrium between proliferation factors and senescence.

The annexin V conjugated to a fluorescent dye is a Ca^2+^-dependent phospholipid-binding protein with high affinity for phosphatidylserine present at the membrane surface of apoptotic cells. In contrast, damaged and dead cells are stained by PI through the exclusion process [39]. Hence, the high cell viability observed through MTT is corroborated by annexin V/PI staining, which showed no toxicity of monocerin until 1.25 mM. MTT targets mitochondria and AO the nucleus. However, the high percentage of viable cells confirmed that monocerin did not affect mitochondrial function in endothelial cells.

Acridine orange is a fluorescent dye extensively used that exhibits metachromatic properties. It is employed to monitor various biological processes, including nuclear fragmentation and pH gradients in biological membranes, as well as to assay apoptosis, autophagy, to measure the adhesion of leukocytes, and as a lysosomal marker [40].

In living cells, acridine orange accumulates mainly in the nucleic acid in double-strand (DsDNA), preferentially in lysosomes and nucleus endosomes, autophagosomes, RNA, and DNA. It also binds to phosphate acids in single strand (ssDNA), RNA, or vacuoles [41]. AO fluoresces bright green in the presence of nucleic DNA. Whereas, in deep red, it is indicative of the presence of RNA (cytoplasm and nucleoli) [42]. Chromatin granules are easily detected with AO staining, and DNA is only partially accessible to AO. When bound to RNA, AO increases the intensity of both red and green fluorescence, and this phenomenon has not been observed for cells treated with monocerin.

Normally, in living cells, the concentrations of bound dye cations are low, producing green fluorescence. In contrast, in damaged or dead cells, yellow or red fluorescence is observed, respectively. The modification of the fluorescence is due to a progressive increase in AO binding by proteins through electrostatic means [43].

AO is a membrane permeable dye that can stain viable and damaged cells, allowing the detection of apoptotic cell death through morphological changes, namely, necrotic and apoptotic stages, mainly in the early and late phases. Viable cells typically exhibit fewer accessible electronegative charges present on proteins. Apoptotic cells, on the other hand, are bright and condensed, with fragmented nucleic acid and intact cell membrane [44].

The endothelial cells were stained with AO, and the nuclei, mainly nucleoli and cytoplasm, exhibited green fluorescence. Orange fluorescence is due to acidified lysosomes and a granular pattern in the cytoplasm. Under monocerin treatment, endothelial cells appeared green with normal nuclei morphology, mainly for lower concentrations. However, orange fluorescence was not observed for the endothelial cells treated with monocerin in any concentration, indicating the absence of early apoptotic stage, as observed by annexin V/PI analysis.

The interior of the mitochondria leads to the accumulation of fluorescent lipophilic cations in mitochondria. Rhodamine 123 is a fluorescent cationic dye that binds to mitochondrial membranes due to its negatively charged environment and inhibits transport processes, especially electron transport, delaying internal cell respiration and accumulating the dye within the mitochondria [45]. This dye is a marker of the energy-supplying metabolic process, and the intensity of its accumulation is, therefore, related to the metabolically active living cells [46].

At 0.625 and 1.25 mM, monocerin-treated endothelial cells had a homogeneous mitochondrial distribution in the cytoplasm.

In this current study, the SEM’s morphological analysis of the HUVECs showed dead cells after apoptotic mechanism activation due to treatment with monocerin at a high concentration of 1.25 mM.

Although we have demonstrated the endothelial cells’ morphology after exposure to mycogenic silver nanoparticles and evident cell–cell interactions with the presence of several microvilli on their surface [47], the literature describing the ultrastructural arrangement and morphology of endothelial cells by SEM are scarce, and consequently, it is difficult to compare the monocerin effect with other compounds. In spite of that, SEM analysis indicates that plasmatic membrane alterations during apoptosis are mainly due to biochemical effects, not involving morphological damage, except by the microvillus loss with plasmatic retraction observed on the cells mainly when treated with monocerin at 1.25 mM. These are the main characteristics of apoptosis, distinguished by the apoptotic bodies and cellular membrane degradation.

Nevertheless, the induction of apoptosis can be through programmed cell death, or by proliferation, which is a process caused by pro-apoptotic proteins, mostly caspases that can incite proliferation arrest. Both mechanisms can replace dying cells and are beneficial for the organism, allowing tissues to quickly eliminate damaged or potentially dangerous cells and replace them with healthy neighbors’ progeny.

Granules adhered to the cell membrane of HUVECs in the control group observed by SEM were significantly modified due to treatment with monocerin, mainly at higher concentrations. This result corroborates the cell viability and senescence that shows fragmented DNA, which is, however, not different from the control.

## 4. Conclusions

The fungal strain used in this study was successfully identified as the filamentous fungus *E. rostratum*. Monocerin was not cytotoxic to endothelial cells, maintaining at least 83.33% of cell viability even at the high concentration of 1.25 mM for 24 h. This high cell viability indicates that the metabolic function of lysosomes and mitochondria were maintained even at high concentrations of monocerin. Monocerin-induced cell proliferation is noteworthy with regard to ultrastructural analysis. SEM confirmed no senescence and apoptosis on endothelial cells. The data represent a significant contribution to help elucidate the mechanism of action of monocerin at the cell level for future application, such as in the pharmaceutical area for challenge-requesting cell proliferation, such as in regenerative medicine.

## 5. Material and Methods

### 5.1. Materials, Chemicals and Cell Line

HUVECs were obtained from the American Type Culture Collection (ATCC^®^ CRL-1730™, Manassas, VA, USA).

Penicillin, streptomycin, fetal bovine serum (FBS), and RPMI 1640 were purchased from Cultilab (Campinas, SP, Brazil). Potato dextrose (PD) broth and PD agar (PDA) were purchased from Himedia (Mumbai, India). Dimethyl sulfoxide (DMSO) (Sigma-Aldrich #276855, Saint Louis, MO, USA), 5(6)-carboxyfluorescein diacetate *N*-succinimidyl ester (CFSE), X-gal, trifluoroacetic acid (TFA), and MTT were purchased from Sigma-Aldrich (Saint Louis, MO, USA). The Annexin-V-FITC Kit was obtained from Molecular Probes (#V13241). All other chemical reagents were of analytical grade or, in specific cases, described in the text.

### 5.2. Isolation and Taxonomical Identification of the Fungal Strain

Stem and leaves of the bush *C. blanchetianus* were collected from the *Caatinga* biome in April 2010, and, after being carefully stored in an aseptic bag, they were transported to the laboratory. An endophytic fungal strain, coded as FV3, was isolated from the leaves, according to the method previously described [48]. Briefly, after careful surface disinfection, one leaf was cut into small fragments (4–6 mm), which were placed onto a Petri dish containing PDA and tetracycline (100 µg/mL). After incubation at 28 °C for three to seven days, the hyphal tips of each morphologically different mycelium that emerged from the leaf fragments were sub-cultured until the obtaining of a pure colony for later identification. The species identification was performed by the amplification of the ITS region of rDNA using ITS1 and ITS4 primers [49]. Briefly, the fungus was grown on PDA (Himedia M096), and the mycelia were macerated with liquid nitrogen. Genomic DNA was extracted with the Wizard Genomic DNA purification Kit^®^ (Promega, Madison, WI, USA), according to the manufacturer’s instructions. The quality of the extracted DNA was evaluated in 1.5% agarose gel. ITS amplification was carried out using the primers ITS1 (5′ TCCGTAGGTGAACCTGCGG 3′) and ITS4 (5′ TCCTCCGCTTATTGATATC 3′), following a PCR program of 94 °C for 1 min and 30 s, 35 cycles of 94 °C for 35 s, 55 °C for 2 min, 72 °C for 45 s, 72 °C for 10 min and held at 4 °C. After purification of the PCR products with the QIAquick PCR purification kit (Qiagen, Valencia, CA, USA), the amplicons were sequenced on an ABI 3730 DNA analyzer (Applied Biosystems, Foster City, CA, USA) using a BigDye Terminator v3.1 Cycle sequencing kit (Applied Biosystems). Sequences were aligned and compared to records in the NCBI (National Center for Biotechnology: http://www.ncbi.nlm.nih.gov (accessed on 1 January 2020)) using BLAST (Basic Local Alignment Search Tool). The best hits of well characterized strain deposited in GenBank were retrieved from the databases and subsequently used for alignment and phylogenetic analysis with Molecular Evolutionary Genetics Analysis (MEGA) software version 6.0. The evolutionary history was inferred through the neighbor-joining method, and evolutionary distances were computed using the Jukes-Cantor method. After taxonomic identification, the fungus strain was deposited at the Adolfo Lutz Institute Collection (IAL 7247) (http://www.wfcc.info/ccinfo/collection/by_id/282 (accessed on 3 February 2023); http://www.ial.sp.gov.br/ (accessed on 3 February 2023)), São Paulo, Brazil.

Furthermore, the fungal cell morphology was analyzed using scanning electron microscopy (SEM) and transmission electron microscopy (TEM).

### 5.3. Morphological Analysis of the Fungus E. rostratum

#### 5.3.1. Microcultive of the Fungus Strain and Analysis by Optical Microscopy

The fungal strain was cultured in PDA at 28 °C for five days on a slide covered with coverslips. The coverslips were collected and stained with 200 µL of lactophenol cotton blue stain. The sample was analyzed using an optical microscope (Olympus BX 41), coupled with a digital camera (Olympus SC30) and the image software cellSens software version 4.1.

#### 5.3.2. Morphological Analysis by Scanning Electron Microscopy (SEM)

The fungus *E. rostratum* was grown in a Petri dish with PDA at 28 °C for 15 days, and small fragments of the mycelia were processed [50]. Briefly, the SEM samples were fixed in Karnovsky’s solution for 5 h, and then they were subjected to a vacuum for fixation for another 3 h [51]. The sample was impregnated with 1% osmium tetroxide for 1 h and dehydrated with a gradient of ethyl alcohol. The samples were deposited on a copper sample holder covered with parlodium film (Leica EM CPD030 model, Leica Microsystems, Buffalo Grove, IL, USA), and metallization with a gold bath was performed using the Sputtering system (Leica EM SCD050 Model, Leica Microsystems, Illinois, USA). The fungus morphology was analyzed using a FEI QUANTA 250 SEM.

#### 5.3.3. Morphological Analysis by Transmission Electron Microscopy (TEM)

For TEM analysis, the fungus *E. rostratum* was grown in a Petri dish with PDA at 28 °C for seven days, and small fragments of the mycelia were processed according to specific procedures, described by Heymann et al. [52].

Shortly after this, the mycelium, free of residues from the culture medium, was fixed with a 2.5% glutaraldehyde and 2% paraformaldehyde solution in a 0.1 M cacodylate buffer at pH 7.2 at room temperature for about 12 h. Afterwards, the sample was washed with 0.1 M sodium cacodylate buffer and pH 7.2. Post-fixation was performed in 1% osmium tetroxide (OsO4) in 0.1 M cacodylate buffer and pH 7.2. The samples were pre-soaked in pure resin at room temperature for about 12 h (overnight) and kept in a vacuum chamber at 60 °C for 48 h. The samples were analyzed with TEM (Leo 906 E Zeiss # serial number 9682, 120 Kv), coupled with the software iTEM for image capture.

### 5.4. Fungus Culture and Purification of Monocerin

The fungus was cultivated in PDA at 28–30 °C for seven days, and all the colonies of a Petri dish (60 × 15 mm) were transferred to a 2 L Erlenmeyer containing 500 mL of PD. The culture was kept for 15 days at 28 °C and 150 rpm. The biomass was removed through filtration in cheesecloth, and the supernatant was submitted to solid phase extraction (SPE) in a C18 cartridge (Speed SPE Cartridges—Octadecyl C18/18%) using methanol at 20 and 100% as eluent, resulting in fractions SPE20 and SPE100, respectively. The fraction SPE100 was dried with rotaevaporation (Bucchi© R-210, Flawil, Switzerland) and purified through high performance liquid chromatography (HPLC) using a C18 column (Ace^®^, 250 × 4.6 mm) with the isocratic method in two steps around 40–56% of mobile phase B, according to the impurities present (mobile phase A: trifluoroacetic acid (TFA) 0.1%; B: water/methanol/TFA 90:9.9:0.1%), leading to the obtaining of pure monocerin. The chemical structure of monocerin was determined by proton and carbon nuclear magnetic resonance (^1^H-NMR, ^13^C-NMR) (Agilent 500/54 premium shielded) and EIMS, as previously described [6,7,15], and the purity was confirmed by gas chromatography-mass spectrometry (Agilent GC/MS, 7890/5975C).

### 5.5. Biological Assays

#### 5.5.1. Monocerin Preparation

Monocerin was dissolved in DMSO for a final concentration of 415 mM, sterilized through filtration in a 0.22 µm membrane (cellulose regenerated), and kept frozen at −20 °C in small aliquots of 100 µL. Immediately before the use, the aliquots were diluted to 10 mM in phosphate-buffered saline (PBS, pH = 7.2), and, then, from 1.25 to 0.02 mM in serum-free RPMI-1640 medium. The final concentration of DMSO in the assay was less than 0.005 to 0.3% for the lower and higher concentration of monocerin at 0.02 and 1.25 mM, respectively. It is known that, in this concentration range, DMSO is not cytotoxic for HUVEC [53]. For all assays, control cells received serum-free RPMI-1640 medium.

#### 5.5.2. Cell Culture

HUVECs were cultured in RPMI-1640 medium supplemented with 10% FBS, penicillin (100 UI/mL), and streptomycin (0.1 mg/mL) in a humidified incubator at 37 °C with 5% of CO_2_ until 80% confluence. The cells were then trypsinized, neutralized with FBS, and the concentrations were adjusted with serum-free RPMI-1640 medium according to the protocol of each assay.

#### 5.5.3. Cell Viability—MTT Assay

HUVECs (1 × 10^5^ cells/well) in a 96-well flat-bottom microplate were treated with monocerin from 0.02 to 1.25 mM in six replicates for 24 h, and the cytotoxicity was evaluated with the MTT assay [54]. Assays were performed at three different times, and the cell viability was expressed by the percentage of viable cells compared to untreated cells, used as control.

After treatments, the culture supernatant was removed, and 100 µL of MTT at 0.5 mg/mL in serum-free RPMI-1640 medium without phenol red were added to each well, and the plates were incubated for four hours. After removal of the supernatant, the resulting intracellular formazan was solubilized with 100 µL of isopropanol/HCl 0.1 M under shaking, and the absorbance was determined at 570 nm in a microplate reader (SpectraMax 190, Molecular Devices, LLC, Sunnyvale, CA, USA).

The cellular viability was expressed by percentage and comparison with untreated cells (control) considered as 100% of cell viability. The concentration of monocerin that inhibited cell growth by 50% was defined as the IC50.

Data were calculated using GraphPad Prism 6.0 software and analyzed with One-way ANOVA / Dunnett’s multiple comparison tests (between control and treated cells).

#### 5.5.4. Senescence Associated β-Galactosidase (SA-β-gal) Activity

Cytochemical staining in situ for senescence-associated β-galactosidase (SA-β-gal) was performed, as previously described [55], using the senescence cell histochemical staining kit, according to the manufacturer’s instructions (Sigma #CS0030). Briefly, HUVECs at 1 × 10^5^ cells/well in a 12-well plate were treated with monocerin at 0.02, 0.15, 0.625, and 1.25 mM in triplicate for 24 h. Untreated cells received only serum-free RPMI-1640 and were used as control.

After treatments, the cells were carefully washed with PBS, fixed with 0.6 mL of buffer (Sigma #F1797) for 6–7 min at RT, and incubated with β-galactosidase substrate staining solution at 37 °C for 12 h. The staining solution was removed, and the cells were covered with glycerol (70%) and kept at 4 °C until the analysis. Stained cells were observed in an inverted light microscope (Olympus CKX41) with magnification of 200×. A digital camera (Sony DSC 300) was used to record micrographs of three representative fields of each monocerin treatment and of the control.

The number of senescent (positive to SA-β-gal and represented by a blue color) and non-senescent cells (negative to SA-β-gal and represented as uncolored) under bright field illumination were counted. The percentage of senescence was calculated for monocerin treatments compared to the untreated cells (control). Data were calculated using GraphPad Prism 6.0 software and analyzed with One-way ANOVA/Dunnett’s multiple comparison tests (between control and treated cells).

#### 5.5.5. Annexin V/Propidium Iodide Apoptosis Assay

To evaluate if monocerin was able to induce apoptosis in HUVECs, an isothiocyanate (FITC)-annexin-V/PI apoptosis detection kit was used, according to the manufacturer’s recommendations, followed by flow cytometry analysis.

Cells were exposed to monocerin in a six-well plate (4 × 10^5^ cells/well) at 0.02, 0.15, 0.625, and 1.25 mM for 24 h at 37 °C/5% CO_2_, in six replicates in two independent experiments. Untreated cells were used as control and received serum-free RPMI-1640 medium. PI was added to distinguish between early apoptotic (annexin V^+^/PI^−^) and late apoptotic or necrotic (annexin V^+^/PI^+^) cells. After the treatments, cells were carefully processed and analyzed by using flow cytometry analysis (FACSCalibur^®^, Becton-Dickinson (BD) Immunocytometry Systems, San Jose, CA, USA) using emission filters of 515–545 nm for FITC (green) and 600 nm for PI (red). A total of 10,000 events were acquired for each sample. Data were analyzed using the standard Cell Quest software, and the percentage of cell membrane integrity was calculated from the ratio of the number of membrane-intact cells to the total number of cells.

#### 5.5.6. Analysis of Endothelial Cells Proliferation

The cellular proliferation through the CFSE labeling method was performed, as previously described [56]. The cells were labeled with CFSE (1 µL at 5 µM for 1 × 10^6^ cells/mL) and seeded at 1 × 10^5^ cells/well in a 12-well plate. After overnight incubation, the cells were treated with monocerin at 0.02 and 0.15 mM, in triplicate, for 24, 48, and 72 h. The cell division rate was determined by using flow cytometry analysis (FACSCalibur^®^, BD Immunocytometry Systems, San Jose, CA, USA), collecting 10,000 events for each sample. The data were analyzed using the ModFitLT 3.2 software, and the proliferation index of two independent assays was calculated using GraphPad Prism 6.0 software. Statistical analysis was performed with one-way ANOVA/Tukey´s multiple comparison tests.

#### 5.5.7. Morphological Analysis of the Endothelial Cells by

##### Scanning Electron Microscopy (SEM)

HUVECs were distributed on coverslips in a 24-well plate (2 × 10^5^ cells/mL), and, after overnight incubation at 37 °C and 5% CO_2_, they were exposed to 500 µL of monocerin at 0.02, 0.15, 0.625, and 1.25 mM or to RPMI-1640 medium as control, for 24 h. Cells were carefully processed, covered with a gold film, and analyzed with laser SEM (FEI QUANTA 250) at an accelerating voltage of 10 kV [51]. The modification or ultrastructure arrangement of the cell morphology was observed, and the images were obtained through secondary electron analysis [52].

##### Laser Scanning Confocal Microscopy

The morphological analysis of HUVECs treated with monocerin was performed using the lysosomotropic fluorochrome acridine-orange dye (AO—3,6-dimethylaminoacridine) [57] and the fluorescent dye rhodamine 123 that binds to metabolically active mitochondria, as previously described [58].

In brief, HUVECs were distributed on coverslips in a 24-well plate (1 × 10^4^ cells/well) and incubated overnight at 37 °C/5% CO_2_. The cells were treated with 500 µL of monocerin at 0.02, 0.15, 0.625 and 1.25 mM, in triplicates, for 24 h at 37 °C/5% CO_2_. Untreated cells received serum-free RPMI-1640 medium and were assigned as control, while the media without cells were assigned as blanks. Cells were washed with PBS and incubated with 20 µL of rhodamine 123 (0.7 mg/mL) for 30 min at 37 °C or 10 µL of AO (10 mg/mL in PBS FluoroQuench AO/EB Molecular Probes, USA) for 10 min in the dark. After incubation, the cells were washed with PBS, and the coverslips were carefully mounted onto microscope slides and analyzed using confocal microscopy laser LSM 510 META (Zeiss, Oberkochen, Germany).

Images for AO were recorded at 200–400 Hz using line averaging of 4–8, and optimal *Z*-stack slice intervals were calculated by the Leica Application Suite [44]. For rhodamine 123 dye, the λ_EXC_ and λ_EM_ were 530 nm and 610 nm, respectively. Representative fields were recorded with identical shutter speeds using a 1600 ASA Kodak Ektachrome color positive film, and cell morphology considering the lysosomal content was analyzed.

### 5.6. Statistical Analysis

The results are expressed as mean ± SE and statistically analyzed using GraphPad-Prism 6.0 software with One or Two-way ANOVA/Tukey’s or Dunnett´s multiple comparison tests. Differences concerning the control values were considered statistically significant when *p* < 0.05.

## Figures and Tables

**Table 1:** Annexin V/PI labeling of untreated (control) endothelial cells or after treatments with monocerin (mean ± SE) (*n* = 12).

Monocerin (mM)	Viability	Necrosis	Initial Apoptosis	Late Apoptosis
Control	93.1 ± 0.0	1.4 ± 0.0	4.6 ± 0.0	0.9 ± 0.0
0.02	94.3 ± 0.8	1.2 ± 0.2	4.0 ± 0.6	0.6 ± 0.2
0.15	92.7 ± 0.3	2.3 ± 0.2	4.5 ± 0.3	0.5 ± 0.1
0.625	91.9 ± 0.8	3.9 ± 0.5	3.6 ± 0.3	0.6 ± 0.3
1.25	91.5 ± 0.5	2.2 ± 0.1	3.6 ± 0.3	0.5 ± 0.1

## Data Availability

The data used to support the findings of this study are included in the article.

## Supplementary Materials

The following supporting information can be downloaded at: https://www.mdpi.com/article/10.3390/toxins15050344/s1, Figure S1: Profile of the monocerin (2S,3aR,9bR)-6-hydroxy-7,8-dimethoxy-2-propyl-2,3,3a,9b-tetrahydro-5H-furo[3,2-c]isochromen-5-one spectrum by gas chromatography-mass spectrometry (GC/MS, Agilent 7890/5975C). Table S1: Genbank accession number for sequences used in phylogenetic analysis.
