# Supplementary material for: Effect of Monocerin, a Fungal Secondary Metabolite, on Endothelial Cells
Source: Toxins (Basel). 2023 May 18;15(5):344. doi: 10.3390/toxins15050344 (PMC10222415; doi:10.3390/toxins15050344)
Supplement: Supplementary file 1 [file toxins-15-00344-s001.zip › toxins-2354739-supplementary.pdf]

toxins MDPI

Supplementary Material: Effect of Monocerin, a Fungal Secondary Metabolite, on Endothelial Cells

Tainah Colombo Gomes, Rafael Conrado, Rodrigo Cardoso de Oliveira, Priscila Jane Romano Gonçalves Selari, Itamar Soares de Melo, Welington Luiz Araújo, Durvanei Augusto Maria and Ana Olívia De Souza

Taxonomic identification of the fungus *E. rostratum*

The taxonomic identification of the fungus *E. rostratum* was based on the phylogenetic tree using the ITS-1 and 5.8S-ITS-2 sequences. The nucleotide sequence file is available from the NCBI database (GenBank accession number MK005195) (Table S1).

Table S1. Genbank accession number for sequences used in phylogenetic analysis.

Species name	Strain	Genbank number
Alternaria infectoria	CBS 210.86	NR_131263.1
Alternaria peucedani	CNU 111485	NR_151840.1
Bipolaris brachiariae	CPC 28819	NR_153654.1
Bipolaris clavate	BRIP 12530	NR_147466.1
Bipolaris crotonis	BRIP 15875	NR_147468.1
Bipolaris secalis	BRIP 14453	NR_147472.1
Curvularia americana	UTHSC 08-3414	NR_146239.1
Curvularia hawaiiensis	BRIP 11987	NR_147480.1
Curvularia malina	CBS 131274	NR_147461.1
Curvularia micropus	CBS 127235	NR_157424.1
Curvularia nicotiae	BRIP 11983	NR_147483.1
Curvularia pseudorobusta	SDAU 992347-2	NR_130653.1
Curvularia tropicalis	BRIP 14834	NR_147486.1
Drechslera avenae	CBS 279.31	NR_153967.1
Drechslera brizae	CBS 190.29	NR_153992.1
Exserohilum fusiforme	KUC5012	GQ241279.1
Exserohilum gedarefense	CBS 297.80	NR_155091.1
Exserohilum khartoumensis	CBS 132708	NR_157476.1
Exserohilum longirostratum	BRIP 16078	LT837826.1
Exserohilum mcginnisii	CBS 325.87	KT265237.1
Exserohilum neoregeliae	CBS 132832	NR_157457.1
Exserohilum oryzicola	CBS 502.90	NR_138225.1
Exserohilum pedicellatum	CBS 322.64	NR_157425.1
Exserohilum protrudens	BRIP 14814	KJ415561.1
Exserohilum rostratum	JN711432.1	ITF0706-2
Pleospora gigaspora	EGS37-017	AY329177.1
Pleospora rosae	MFLU 16-0254	NR_157531.1
Pyrenophora semeniperda	haplotype L (no strain given)	GQ168738.1

From the *E. rostratum* IAL 7247 culture, the secondary metabolite monocerin was obtained, purified and physical-chemically characterized. Figure S2 shows the GC-MS spectrum for this compound.

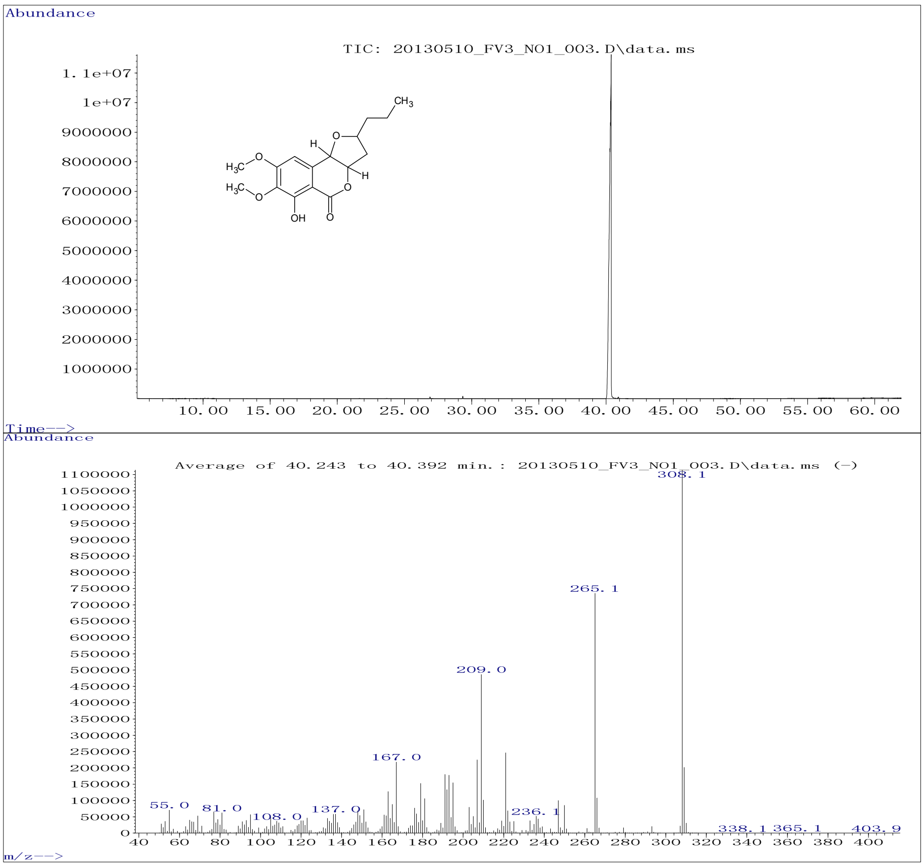

Figure S1. Profile of the monocerin (2S,3aR,9bR)-6-hydroxy-7,8-dimethoxy-2-propyl-2,3,3a,9b-tetrahydro-5H-furo [3,2-c]isochromen-5-one spectrum by gas chromatography-mass spectrometry (GC/MS, Agilent 7890/5975C).
